# Supplementary material for: Bedshift: perturbation of genomic interval sets
Source: Genome Biol. 2021 Aug 20;22:238. doi: 10.1186/s13059-021-02440-w (PMC8379854; doi:10.1186/s13059-021-02440-w)
Supplement: Supplementary file 1 — Additional file 1 Supplemental figures. [file 13059_2021_2440_MOESM1_ESM.pdf]

Additional file 1

Additional file with supplemental tables and figures for:

Gu A, Cho HJ, Sheffield NC. Bedshift: Perturbation of genomic interval sets. *Genome Biology*. 2021.

parameter set	add	drop	shift
add1	0.1	0.0	0.0
add2	0.2	0.0	0.0
add3	0.3	0.0	0.0
drop1	0.0	0.1	0.0
drop2	0.0	0.2	0.0
drop3	0.0	0.3	0.0
shift1	0.0	0.0	0.2
shift2	0.0	0.0	0.5
shift3	0.0	0.0	0.8
add_drop1	0.1	0.1	0.0
add_drop2	0.1	0.2	0.0
add_drop3	0.1	0.3	0.0
add_drop4	0.2	0.1	0.0
add_drop5	0.2	0.2	0.0
add_drop6	0.2	0.3	0.0
add_drop7	0.3	0.1	0.0
add_drop8	0.3	0.2	0.0
add_drop9	0.3	0.3	0.0
shift_drop1	0.0	0.1	0.2
shift_drop2	0.0	0.1	0.5
shift_drop3	0.0	0.1	0.8
shift_drop4	0.0	0.2	0.2
shift_drop5	0.0	0.2	0.5
shift_drop6	0.0	0.2	0.8
shift_drop7	0.0	0.3	0.2
shift_drop8	0.0	0.3	0.5
shift_drop9	0.0	0.3	0.8
add_shift1	0.1	0.0	0.2
add_shift2	0.1	0.0	0.5
add_shift3	0.1	0.0	0.8
add_shift4	0.2	0.0	0.2
add_shift5	0.2	0.0	0.5
add_shift6	0.2	0.0	0.8
add_shift7	0.3	0.0	0.2
add_shift8	0.3	0.0	0.5
add_shift9	0.3	0.0	0.8

Table S1: ***Parameter combinations used in the analysis.***

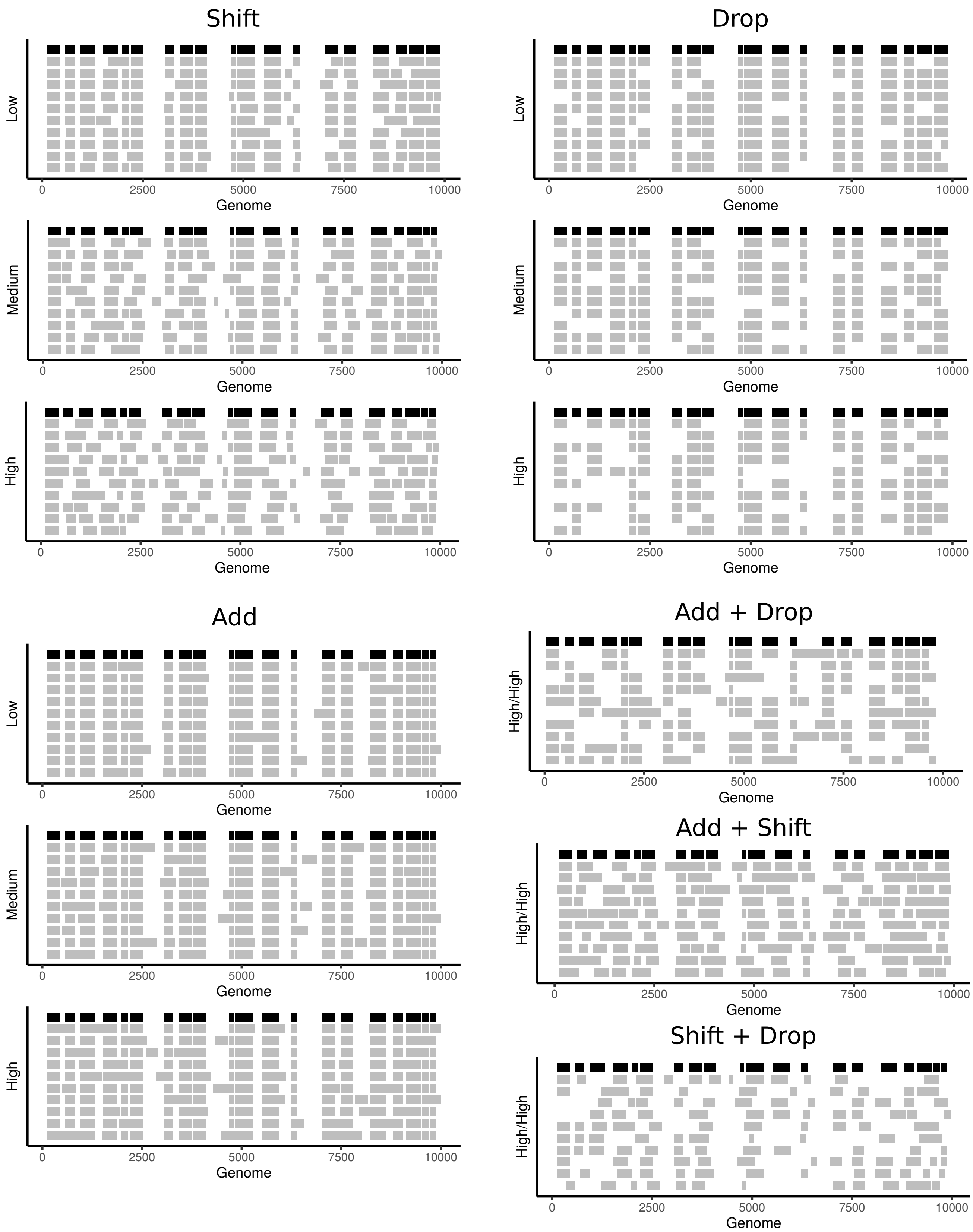

Figure S1: ***Demo visualization of perturbations***. *Original file is shown in black, randomizations in gray. The vertical axis labels depict perturbation degree, corresponding to parameter values in the parameter table. Shift plots are reproduced here from figure 1 for comparison. For combinatorial perturbations, only the maximum perturbation is shown.*

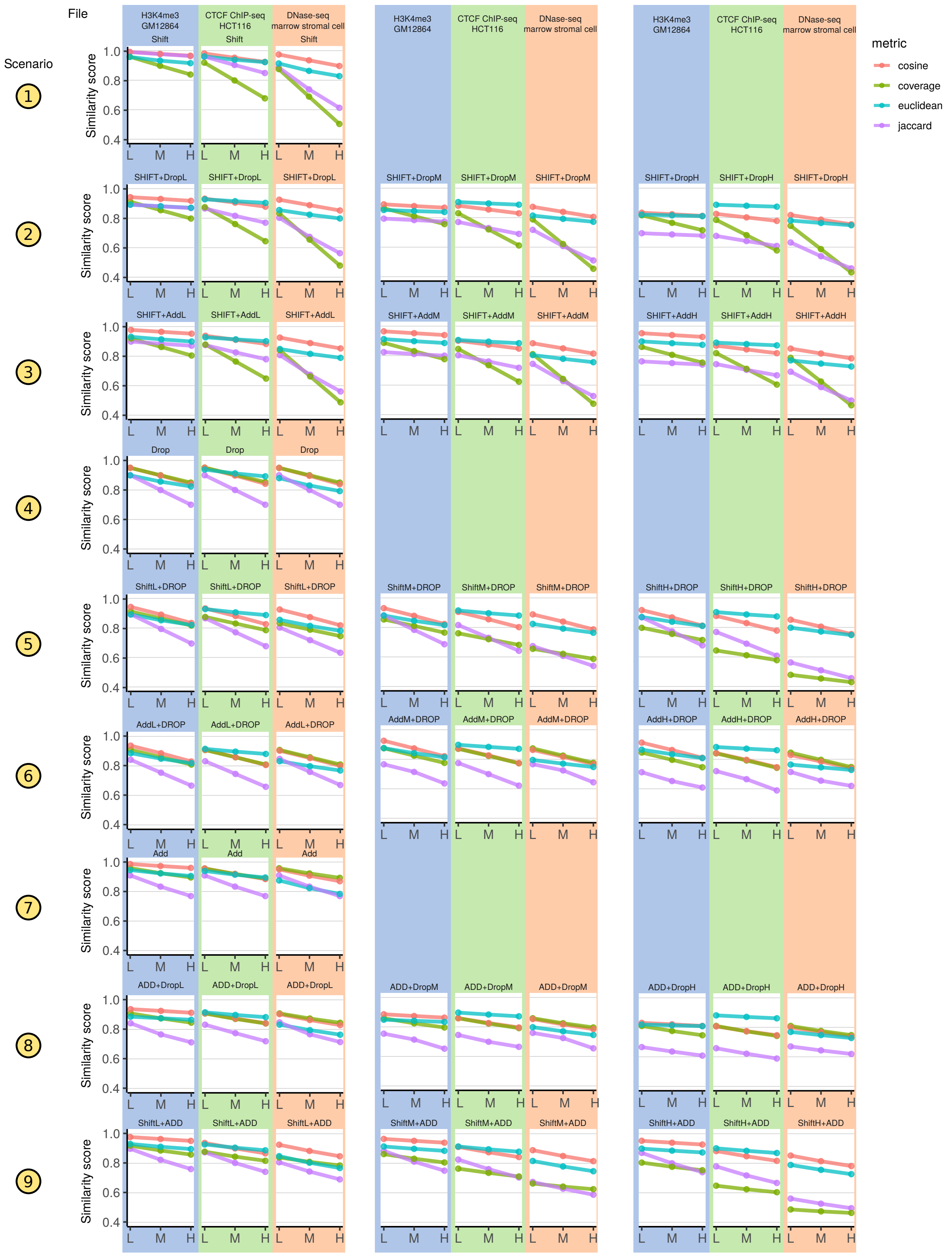

*Figure S2: **Detailed results showing how metrics vary by query file.** Universe is held constant.*

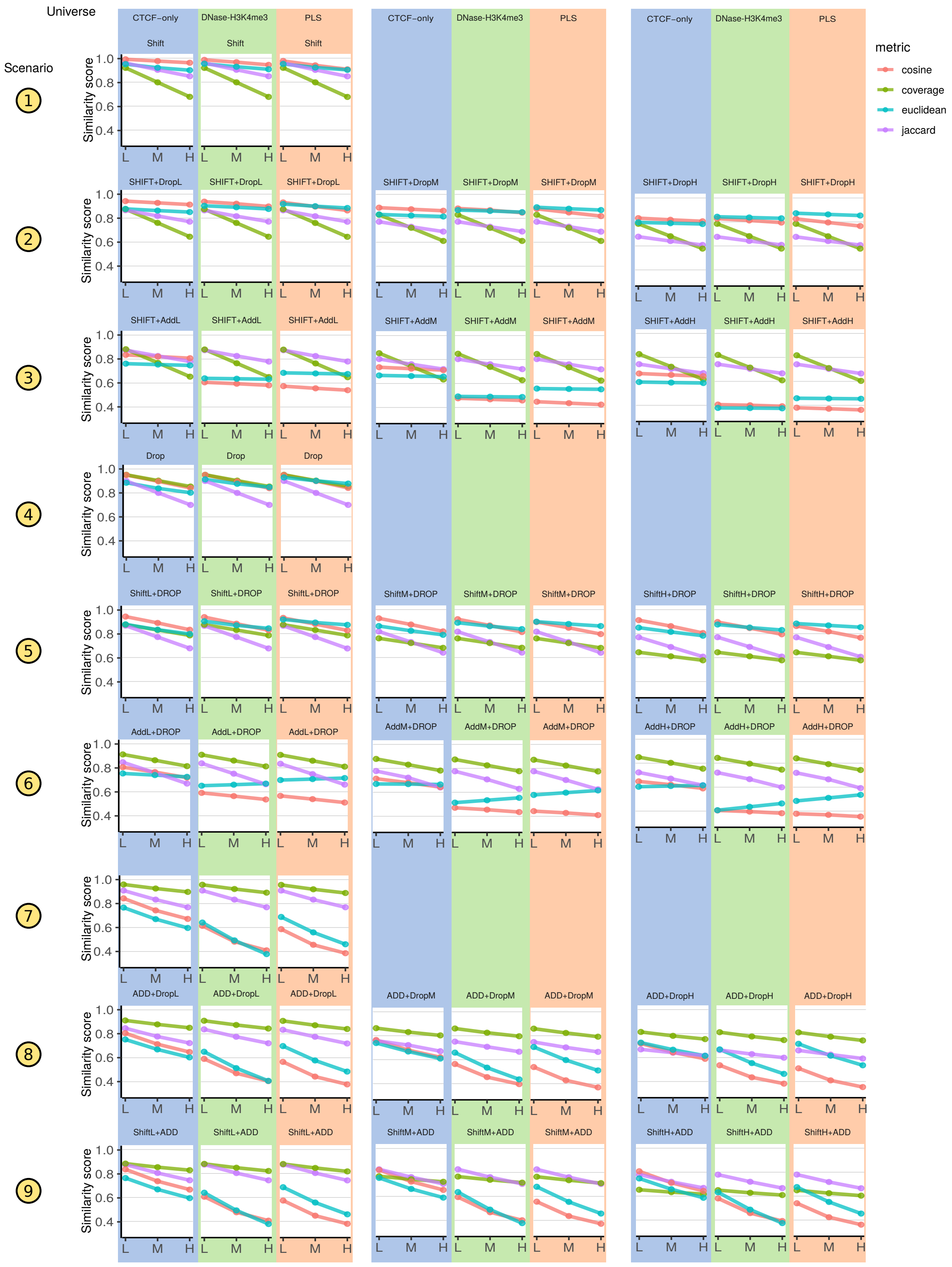

Figure S3: ***Detailed results showing how metrics vary by universe.*** *Query file is held constant, and is the same as in the original study.*
