# Supplementary material for: Bedshift: perturbation of genomic interval sets
Source: Genome Biol. 2021 Aug 20;22:238. doi: 10.1186/s13059-021-02440-w (PMC8379854; doi:10.1186/s13059-021-02440-w)
Supplement: Supplementary file 2 — Additional file 2 Review history. [file 13059_2021_2440_MOESM2_ESM.docx]

Review History

**First round of review**

**Reviewer 1**

**Are you able to assess all statistics in the manuscript, including the appropriateness of statistical tests used?**

Yes, and I have assessed the statistics in my report.

**Comments to author:**

This short report introduces a simple python tool for creating perturbed bed files with a desired percent of regions added, dropped, and/or shifted. The authors then show how various similarity metrics differ in their sensitivity to these changes. While Bedshift makes modifying bed files more convenient, neither the paper nor the tool provides direction in how it should be used to test realistic hypotheses. Beyond this, there appears to be a fundamental error in how new regions are added. The authors should not have seen additional regions added to have significant overlap with regions dropped or shifted away, because the genome is normally extremely large compared to the region set. But because no methods were provided describing how this is done, I could not assess why. The authors state that the importance of this tool is that it produces region sets with known, ground truth differences. However, the differences that it produces are not realistic, and thus likely do not mimic the types of differences seen in real world experimental region sets. This is because the perturbation types offered do not take into account any properties of genomes, such as tendency of regions to occur in promoters, enhancers, genes, and not to occur in centromeres and other closed chromatin areas. Overall, the tool was simplistic, fairly easily programmed independently by a bioinformatician and yet not easily used properly by a biologist.

Major concerns:

1. There are many other additional options the authors could have added to make this tool more practical. However, as is, it is quite simplistic and not difficult for bioinformaticians to program themselves. If it is meant for non-programmers, this does not work either, because there are no functions provided to do later steps in a comparison (e.g. generating 100's of permutated files, comparing, and interpreting results). Thus, some programming is required. The authors also do not demonstrate how their tool would be used in a real situation with a specific hypothesis.

2. The authors say that the region set comparison methods use Jaccard, coverage, or Fisher's exact test. I'd like to see the authors implement their approach with some of the full, previously published region set comparison methods mentioned in the Introduction, in addition to the simple distance methods.

3. A major limitation is that there is no ability in the program to specify general location of the added or dropped regions. For example, how should added regions be distributed among promoter regions, Enhancers, and Introns? No genomics experiment produces a region set that is perfectly distributed randomly across types of genomic regions. Thus, to avoid spurious similarities due simply to two sets both being overrepresented in promoters, for example, there should be a way to handle this.

4. Methods for how regions are added, etc. are lacking. There should be a Methods section for this. From what total set of chromosomes and positions are regions added? Are all possible regions added with equal probability? Does the user need to input chromosomal lengths for the genome? Knowing this would allow understanding what happened with #7 below. Maybe the authors are alluding to this in the Euclidean distance section of Methods when they talk about the "universe", but this is not clear, and it's an inappropriate place for this, since the "universe" is part of the Bedshift function itself, not just the Euclidean distance.

In addition, in the Results section "Overview of bedshift", the authors mentioned that the shift and add operations both include a random value from a normal distribution. However, why normal distribution was selected and how the parameters (the mean and variance) should be defined were not explained.

5. Authors state "We randomly selected one file from publicly available data from ENCODE (ENCODE Consortium, 2012)." However, as far as I can tell, they do not state which one. Not knowing anything about the properties of this dataset limits my ability to interpret their results. Also, multiple datasets should be used to examine differences in results by dataset. E.g., a dataset with 800,000 genomic regions versus one with only 1,000 regions may behave quite different. Therefore, the conclusions about the similarity metric comparisons may be biased by the characteristics of the original dataset from which all the permutations were generated.

6. In the Shift results section, the authors state "constructing the vectors, which contained regions with 537 bp median length compared to 320 bp median length from the original dataset". There is no explanation for what is meant by "the vectors". Also, if this difference in length was going to be an issue then the authors should repeat the perturbations with the same length as the original dataset for a more direct comparison.

7. The comparison with Fisher's exact test (FET) was superficial. The authors could have performed comparisons with combinations of add, drop, and shift. They also could have assessed -log10 p-values, or plotted log odds ratios instead for better visualization. A question of potential interest for FET is at what point the bed files are no longer significantly overlapped, but readers cannot tell this from Figure S1.

Minor concerns:

8. Authors should point out that their perturbation approach is to identify significant differences between region sets rather than significant overlap (i.e. the null hypothesis is that they are the same). Both hypotheses are of interest for different purposes, but the difference between them may not be clear to many readers.

9. The authors state "once enough regions have been dropped, then adding has an increased probability to add back a region that was previously dropped, causing a canceling effect." While this is theoretically true, the probability of adding a random region of the genome that's the same as one dropped is virtually nil. It would not make enough difference to be observable in the results. Rather than showing a defect in the Jaccard score, this seems to point to a problem in how Bedshift was written or implemented.

10. The parameters used for the shifting in the paper are not provided

11. Typo on page 1, line 60 (should be "calculate")

**Reviewer 2**

**Are you able to assess all statistics in the manuscript, including the appropriateness of statistical tests used?**

Yes, and I have assessed the statistics in my report.

**Comments to author:**

Gu et al present a very interesting short report entitled "Bedshift: perturbation of genomic interval sets". The content appears two-fold: one the one hand, they introduce bedshift, a novel python suite dedicated to randomly perturbing genomic coordinates; and on the other hand the authors carry out a systematic assessment work on four similarity metrics used to compare genomic intervals . The manuscript is well written and easy to read, the various sections are well described. The package per se is easy to install, well-documented on github as well as on the command line, and available from the shell inferface or python.

The work presented there is, in my opinion, far reaching. From what I could extrapolate, bedshift itself represents a much-needed tool that could be used systematically to quantify the significance of overlaps between genomic coordinates from control versus treatment conditions, by randomising the control. Out of experience, two conditions could easily represent such randomised versions of each other; I think this is where this tool comes in. It could also be used to determine random variability between replicates.

The report further sheds light on candidate metrics to use depending on the type of experiment. For example, the shift method could simulate sliding, while the add method could represent external stimulation and the drop method the removal thereof or inhibition.

I only have one concern and some minor comments but I think that the manuscript would benefit from addressing those:

1) Unless I missed it, in terms of reproducibility, did the authors observe similar results (i.e. metric scores) using other bed files chosen at random from ENCODE? It would be useful to have error bars on the metric graphs.

2) As stated above, bedshift could be used as a very powerful randomisation control when comparing e.g. bed files from same extraction method (e.g. same antibody for ChIP-Seq) in two different conditions. I think the manuscript would substantially gain if the introduction and discussion clearly mentioned that it can be used to determine if e.g. variability between two conditions is random or biological. By this, I mean that it could be used by the scientific community to randomise a control file, and then computing e.g. the significance of overlap or any other metric between the randomised control and treatment

3) Page 2, lines 47-49, "These parameters were chosen arbitrarily to be not too small as to not have significant change, and not too large as to be completely different".-Further to the previous point, I think it would be useful to present some level of significance associated with the metrics, e.g. with hypergeometric tests.

4) From the package documentation, bedshift -s and -a seem to present mean and stdev shift and add options. From the materials and methods, it is unclear by how much regions are shifted and added in analysis. Adding full settings of perturbations, with values for e.g. --shiftmean, --shiftstdev, --addmean, --addstdev in a supplementary methods section would benefit the study in terms of clarity. The detailed parameters of bedtools coverage should also be incuded. Also the details of overlap methods used to compute the Jaccard similarity index could be useful to have, i.e. were overlaps defined as total or partial overlap for a given region, if partial what was the minimum allowed?

5) In the shift method, did the authors take into account the average distance between regions, as excessive shift could result in overlap if greater than the average distance between regions?

6) Page 4, column 2, lines 25-26 "was constructed by merging regions from 300 BED files in the ENCODE dataset ". Can the authors comment on why they chose 300 experiments from ENCODE as universe and not e.g. the ENCODE regulatory database, or O'RegAnno or ReMap to name a few? Also, if they could clarify whether these were chosen randomly or not, and if not, what kind of experiments were chosen (TF ChIP-Seq, DNase-Seq, or ATAC-Seq?). Ideally they should not include broad histone peaks as these are very large.

7) On figure 2 and the associated methods, I could not determine how the combinations of operations were carried out, for example for shift+drop, is the drop carried out before or after shifting (I would assume after, but could not find confirmation in the text), could the authors clarify?

8) While most bed files will be unstranded, the rising number of e.g. CLIP/FLASH-Seq (which can be stranded) experiments as well as of e.g. eRNA or repeat element genomic regions (also potentially stranded) might warrant the implementation of a strandedness parameter in bedshift; alternatively the authors could discuss this limitation.

9) Add+dropH and drop+addH seem to show recovering jaccard distance: this implies that regions added at random would partially reconstitute the original file. Is this plausible? And if so could this be prevented via a parameter?

10) For the shift method, the authors indeed mention that decreased coverage is expected, however can they further comment on why does add+shiftH result in increased coverage but add+drop and drop+add result in increased jaccard distance?

**Authors Response**

**Point-by-point responses to the reviewers’ comments:**

Reviewer #1

This short report introduces a simple python tool for creating perturbed bed files with a desired percent of regions added, dropped, and/or shifted. The authors then show how various similarity metrics differ in their sensitivity to these changes. While Bedshift makes modifying bed files more convenient, neither the paper nor the tool provides direction in how it should be used to test realistic hypotheses.

*We apologize for the lack of clarity for direction in how it should be used. We have revised the introduction to try to better communicate the point of this. Bedshift sits upstream of a specific biological use case; its goal is not to test specific dataset, like an experimental ChIP-seq dataset, but to provide a tool to aid in development of methods for comparing genomic interval sets. In fact, we built it because we need it in our own studies of genomic interval comparisons; but its application is at the level of testing how these metrics behave fundamentally. The “realistic hypotheses” that bedshift is geared toward would be something like this: “We propose that comparing two region sets with a Jaccard-based score will rate them as more similar than using a coverage-based score, if the primary difference is that regions are shifted by small amounts”. This would be relevant, for example, if we were comparing replicates of a ChIP-seq experiment and wondered which similarity metric would be most able to capture differences likely to show up in that type of experimental replication.*

*For many years, we have been engaged in analyses of region data, such as comparing chro¬matin accessibility among cell types, comparing DNA methylation patterns among patients, etc. We inevitably reduce these down to collections of genomic regions, or region sets, and we find that we want to learn more about what the regions in these experimental datasets have in common. One approach we’ve used with success is region set enrichment analysis, where we identify pre-existing region sets that are similar to our newly created region set. In hundreds of studies, this type of analysis has yielded novel insight into the biology of a novel region set.*

*But we’ve also ways a bit dissatisfied in the way this analysis works, because there hasn’t been sufficient effort to understand how different region set similarity metrics behave. What does it mean for two region sets to be similar? Bedshift presents a novel perspective on this problem by providing a way to mathematically randomize a region set in thousands of iterations, thereby providing a “known” distance to an existing file. So, in a sense, the “realistic hypotheses” bedshift will be used to test is exactly what we’ve done in this paper: we contend that a region set distance should be greater for region sets that have been perturbed more – but that the degree will vary by distance metric and by type of difference between the region sets. We found it interesting that the different metrics behave differently depending on the type of perturbation. This has implications for how the enrichment-type analyses work, because different metrics will pull out relationships of different types.*

*We hope this explains a bit better the rationale behind bedshift. While it likely has other applications, particularly after many improvements to address your excellent suggestions, we thought of it as most useful to developers of genomics methods who are trying to make those analyses more accurate. We added some text in the introduction to clarify this point.*

Beyond this, there appears to be a fundamental error in how new regions are added. The authors should not have seen additional regions added to have significant overlap with regions dropped or shifted away, because the genome is normally extremely large compared to the region set. But because no methods were provided describing how this is done, I could not assess why.

*Thank you for raising your concern with the interaction between adding and dropping re-gions. We did not adequately explain that the order of the perturbations, when run with all perturbations, is shift, add, then drop. Since regions are added before the drop per-turbation occurs, the drop perturbation may drop regions that were just added (if it were the other way around, then there indeed would be a low probability that added regions replace the dropped regions!). We have updated the paper to highlight the importance of the order of perturbations. We also added a new configuration option, allowing users to specify the order of operations, which we explain in detail below. Finally, we re-ran the whole analysis in a more much controlled system, and the result in question has gone away in our main analysis, which likely reflects that our universe is more appropriate than the previous one. But this observation is now present in a new analysis where we explored the role of different universes, but only for Euclidean distance. This is an interesting new result which can be explained, and also highlights the importance of choice of appropriate universe. We have explained this new result in detail in the revision.*

The authors state that the importance of this tool is that it produces region sets with known, ground truth differences. However, the differences that it produces are not realistic, and thus likely do not mimic the types of differences seen in real world experimental region sets. This is because the perturbation types offered do not take into account any properties of genomes, such as tendency of regions to occur in promoters, enhancers, genes, and not to occur in centromeres and other closed chromatin areas.

*Thank you, we appreciate the comment. We understand the concern that the perturbations don’t take sufficient account properties of genomes. Our perspective was from a more mathe¬matical angle. Metrics that are in common use for comparing region sets (e.g. statistical tests, jaccard score, coverage calculations) are not aware of the biases and complexities of genome distributions; instead, they are merely mathematical constructs for comparing two interval sets with some measurable degree of overlap. We are approaching this from the perspective of trying to better understand how these metrics behave when the intervals vary. For this use case, we felt the perturbations were sufficient to compare them at the mathematical level. But your point does bring up a bigger-picture issue that these metrics can likely be improved by being more tailored to distributions of real interval data. This is definitely important, and so we want to address this more fully.*

*As you suggest, there is also clear value in considering the properties of genomes more real-istically. We directly address this concern in three ways: 1) We have added new file-specificity functions to enable more realistic perturbations, which we describe in detail below; 2) We have revised the text to better communicate the above perspective, and to better explain our add-from-file perturbation, which is more realistic than we originally described; 3) We have added a powerful configuration option to mix-and-match perturbations for much more complex com¬bined perturbations, also described in detail below. Together, these changes make it possible to use bedshift to make arbitrarily complex (and much more realistic) perturbations, and we thank the reviewer for this point which has greatly improved the tool.*

Overall, the tool was simplistic, fairly easily programmed independently by a bioinformatician and yet not easily used properly by a biologist.

*We address this point about utility by bioinformaticians and biologists in point #1 below.*

Major concerns:

#1. There are many other additional options the authors could have added to make this tool more practical. However, as is, it is quite simplistic and not difficult for bioinformaticians to program themselves. If it is meant for non-programmers, this does not work either, because there are no functions provided to do later steps in a comparison (e.g. generating 100’s of permutated files, comparing, and interpreting results). Thus, some programming is required. The authors also do not demonstrate how their tool would be used in a real situation with a specific hypothesis.

*Thank for the comment, we have taken this as an opportunity to improve Bedshift in 2 ways: 1) We increased the features, improving the value added by Bedshift; 2) we released some accessory code and new tutorials, which show how to use it for a realistic analysis. We’ll go through each of these in detail below:*

*First, new features include drop-from-file, shift-from-file. These allow a user to specify which subset of regions should be shifted or dropped. These are in addition to our existing add-from-file pertubation, which we explore more fully in this revision. Furthermore, we have made Bedshift much more customizable with the new - -yaml config option, which allows the user to create a yaml file containing multiple perturbations to run. These features make it easy to construct very complex and specific perturbations using a simple, human-readable yaml file. In the original version, you could specify perturbations on the command-line, but if you wanted to do something more complex, like change the order or repeat one type of perturbation multiple times, you had to use the Python interface. In the new version, the user has total control over the type, order, and number of perturbations by writing a simple configuration file. This allows one to, for example, combine multiple add-from-file perturbations with different rates and different files, providing total control over which types of regions are perturbed at what frequency. With a simple parameter, Bedshift produces hundreds or thousands of replicates. With this update, Bedshift provides a powerful tool even for an experienced bioinformatician; it’s much faster to use Bedshift than to re-implement these calculations.*

*Second, we also sought to improve simplicity and utility for downstream analysis to increase the breadth of the audience. Bedshift focuses on generating simulated datasets that can be used in a wide range of downstream tasks, which we do not prescribe to the user. It is true that these downstream tasks will likely require some additional programming, so users will need to be at least somewhat familiar with how they want to use the perturbed data. There are many other tools, such as R, bedtools, etc, which can be employed for these questions, and users of Bedshift are likely to be familiar with these. Nevertheless, we wanted to simplify this as much as feasible given the broad scope of possible downstream analysis, so we added new tutorials on how to do later steps in the comparison, such as producing 100s of permuted files from a table of pre-defined permutations. We provide an example at the Bedshift documenta¬tion site http://bedshift.databio.org/en/latest/evaluation guide/, and highlighted this site in the “Overview of Bedshift” section. This shows how, with a single line of code looper run ..., we can produce thousands of perturbed bed files for downstream analysis, sorted into folders, for each of a list of pre-defined perturbations, which can be customized via a configuration file. We also produced an analysis repository (https://github.com/databio/bedshift analysis), which provides a self-contained demonstration of how to use Bedshift for a real study, includ¬ing configuration files and scripts that can calculate similarity metrics and plot them using R.*

*Together, these improvements make Bedshift both more useful for experienced programmers and more approachable for less experienced programmers. With these updates, we think Bedshift is easy-to-use, feature-rich, and thoroughly tested tool. It can be installed by bioinfor-maticians and used within minutes.*

#2. The authors say that the region set comparison methods use Jaccard, coverage, or Fisher’s exact test. I’d like to see the authors implement their approach with some of the full, previously published region set comparison methods mentioned in the Introduction, in addition to the simple distance methods.

*We have spent some time investigating this now and ran into few conceptual and practical challenges. Most importantly, in line with your point #8 below, the current study is best suited to approaches that compare significant differences, rather than significant overlaps. We would need to re-run the test with a different scale of perturbation for the popular approaches men¬tioned above, which are would be more suitable for a different experimental design. Given the scope of this paper is already very broad, in particular with the new experiments added in response to review to test affect of the input file (see your point #5) and universe choice, we don’t think it’s practical to fit in an additional experiment with another class of metrics that would also require new parameter sets, given that this paper is a short report format and lim¬ited to 2 figures and 1,500 words. We do think that this would be an interesting topic for a follow-up study.*

3. A major limitation is that there is no ability in the program to specify general location of the added or dropped regions. For example, how should added regions be distributed among promoter regions, Enhancers, and Introns? No genomics experiment produces a region set that is perfectly distributed randomly across types of genomic regions. Thus, to avoid spurious similarities due simply to two sets both being overrepresented in promoters, for example, there should be a way to handle this.

*Thank you for bringing up this point, it is true that our explanation of the add perturbation is not clear about how and where the regions should be added. We have clarified this in the text. To address this, we’ve increased the emphasis on our add-from-file perturbation (- -addfile), which was present but not well explained in our original manuscript. The - -addfile option al¬lows the user to specify a file from which regions are added. This file could contain promoters, enhancers, introns, or any other genomic regions of interest that are to be added to a file. Sec¬ond, we have now implemented the complementary drop-from-file (- -dropfile) option which similarly provides a set of regions that are candidates to drop from the file. Third, we have also implemented shift-from-file, which allows a user to specify regions that can be selected for shifting. Finally, we also added a new feature, add-from-valid-regions, which operates like the random add, but restricts the locations of random adding to a set of valid ranges. This approach is somewhere in between the completely random “add”, and the more specific “add-from-file”, which is restricted to a set of specified regions. All of these are now documented in the methods section.*

*These perturbations allow the user to specify a file, which could for example be regions of a particular type (enhancers, promoters, etc). Now, Bedshift will selectively add those regions, or only drop or shift existing regions that overlap with those regions. With these, Bedshift now provides much more control over the scope of perturbations, which will also be more realistic than random adding or dropping. In fact, these perturbations together make it possible for Bedshift to easily generate many replicates of random mixes of different bed files, which could be useful for deconvolution methods, something we hadn’t considered earlier.*

*We also added a robust new system for specifying perturbations using a configuration file. In the original version, you could specify perturbations on the command-line, but if you wanted to do something more complex, like change the order or repeat one type of perturbation multiple times, you had to use the python interface. In the new version, the user has total control over the type, order, and number of perturbations by writing a simple configuration file. This allows one to, for example, combine multiple add-from-file perturbations with different rates and different files, providing total control over which types of regions are perturbed at what frequency. These options can be used multiple times one after another, allowing a high level of configurability in Bedshift experiments. For more details, see the newly added Methods section “Bedshift operations”.*

4.	Methods for how regions are added, etc. are lacking. There should be a Methods section for this. From what total set of chromosomes and positions are regions added? Are all possible regions added with equal probability? Does the user need to input chromosomal lengths for the genome? Knowing this would allow understanding what happened with #7 below. Maybe the authors are alluding to this in the Euclidean distance section of Methods when they talk about the “universe”, but this is not clear, and it’s an inappropriate place for this, since the “universe” is part of the Bedshift function itself, not just the Euclidean distance. In addition, in the Results section “Overview of Bedshift”, the authors mentioned that the shift and add operations both include a random value from a normal distribution. However, why normal distribution was selected and how the parameters (the mean and variance) should be defined were not explained.

*Thank you for pointing out that the methods are lacking. We have updated the Methods sec-tion to include explanations of how each perturbation operates. To address your question specifically, add adds regions randomly along every chromosome with chromosome lengths based on hg38. In the first submission of this paper we hardcoded these lengths into Bedshift; we have now added a feature called - -chromosome-lengths that allows the user to input a .fasta file containing chromosome lengths. In addition, we have highlighted the “add from file” (- -addfile) feature described above. The regions added from this file are chosen at random with equal chance specified by the user.*

*The shift and add methods used to generate the perturbed data both used the default normal distribution, which was N(320, 20) for the length of added regions and N(0, 150) for the dis-tance that regions are shifted. This has been clarified in the Methods section. These are just defaults and can be changed by the user, either operating at the command line, from within python, or using the configuration file.*

5.	Authors state “We randomly selected one file from publicly available data from ENCODE (ENCODE Consortium, 2012).” However, as far as I can tell, they do not state which one. Not knowing anything about the properties of this dataset limits my ability to interpret their results. Also, multiple datasets

should be used to examine differences in results by dataset. E.g., a dataset with 800,000 genomic regions versus one with only 1,000 regions may behave quite different. Therefore, the conclusions about the similarity metric comparisons may be biased by the characteristics of the original dataset from which all the permutations were generated.

*The issue here is related to some of the philosophical discussion above; our original intent in this study was to compare the mathematical features of a few common metrics, so from our perspective, the exact set of regions we considered was mostly arbitrary. Our analysis explored how random perturbations of different types affect similarity metrics differently. As a result, we probably could have just randomly generated a set of intervals and gotten a very similar result; we selected a real data file only to be in the right general scope. The file in question in the original study was wgEncodeAwgTfbsUwHek293CtcfUniPk.narrowPeak, but in response to this point, we decided to re-run all the analysis in a more comprehensive study.*

*You raise a good point that the file of interest could lead to different biases, which we did not investigate in our original analysis. We are also interested in the issue of considering multiple files, but it was a new dimension that we felt was outside the scope of our original study; but in the revision we have added this as a new study. To explore the role of the input file, we tested 3 files of 3 different types (CTCF TF ChIP-seq on human HCT116, K4me3 Histone ChIP-seq on human GM12864, DNase-seq on human stromal cell of bone marrow). In the process, we also switched from hg19 to hg38. We now introduce the primary results using the CTCF file, as before, but added discussion and a supplemental figure to show the behavior of the other input files. Overall, for this analysis, we show that the general behavior of the metrics does not vary by input file type.*

*In this revision, the 3 query files used as the original file, which is then perturbed with Bedshift are all from the ENCODE consortium:*

*1)• CTCF TF ChIP-seq on HCT116 (http://bedbase.org/bedsplash/713f58a6497a9168a326123919672ebe) 2)• H3K4me3 ChIP-seq on GM12864 (http://bedbase.org/bedsplash/0f84fea95b736ec99914bc66e74ab6e0)*

*3)• DNase-seq bone marrow stromal cell (http://bedbase.org/bedsplash/c75ea5133f825d779a02be41a529342e)*

*The 4 universe region sets used in this analysis are from the SCREEN ENCODE database:*

*1)• GRCh38-ccREs (Primary universe, http://bedbase.org/bedsplash/f31d4aa5e499637f28a338d5768e4ad5)*

*2)• DNase-H3K4me3 (http://bedbase.org/bedsplash/87d8e916fc910254aa61d5a1611622b7) 3)• CTCF-only (http://bedbase.org/bedsplash/fd007af75d7d0ef5c2e76d8e94916dcf)*

*4)• PLS (http://bedbase.org/bedsplash/d0b72e9adfeaf3ec37f303f16ad36bc4)*

6. In the Shift results section, the authors state “constructing the vectors, which contained regions with 537 bp median length compared to 320 bp median length from the original dataset”. There is no explanation for what is meant by “the vectors”. Also, if this difference in length was going to be an issue then the authors should repeat the perturbations with the same length as the original dataset for a more direct comparison.

*Thank you for bringing this point up. The vectors are the genomic region representations that we mapped from the universe (which are used in measuring Euclidean distance and Cosine similarity), as described in the Euclidean distance and Cosine similarity Methods sections. Since this is the first mention of these vectors in the paper, we added a reference to the relevant methods section.*

*On the difference in median length, we merely wanted to point out that it caused the reduced sensitivity of Euclidean distance and Cosine similarity scores to shifting. But to more fully address this, we have now added a new analysis that tested the effect of the universe (See supplementary figure S3).*

7.	The comparison with Fisher’s exact test (FET) was superficial. The authors could have performed comparisons with combinations of add, drop, and shift. They also could have assessed -log10 p-values, or plotted log odds ratios instead for better visualization. A question of potential interest for FET is at what point the bed files are no longer significantly overlapped, but readers cannot tell this from Figure S1.

*Indeed, we agree that the FET analysis could be improved. Our original FET analysis was problematic because its range that it could detect similarity in was limited to only shift pertur-bations. For add or drop perturbations, one element of the contingency table would be 0, and the FET is undefined. After considering this further, we have some additional insights. You bring up the question of “at what point the bed files are no longer significantly overlapped”, which is related to your point #8 below and to the issue with the FET. The key point is that FET as a metric is more useful for more distant relationships, relative to the other scores we considered. Consider a BED file with 100,000 regions. If you randomly drop 90% of the re-gions, and then add new non-overlapping random regions, then the contingency table would look something like (a=10,000; b=90,000; c=90,000; d=100,000). This yields a p-value off the chart (< 10−16) – and that’s with a 90% drop. This demonstrates that these region sets, even after what you may consider a substantial perturbation (90% drop and add), are still highly highly unlikely to be independent. The FET tests the null hypothesis that the two sets are independent, and given the large numbers, the p-values quickly saturate.*

*To avoid the confusion, we have elected to remove these results from the paper, given that it is way too long already with the above analysis and the new experiments we have added to test affect of the input file and universe. It would be interesting to do an alternative study starting from 2 different files and slowly introducing similarity to test the other class of metrics, like FET, which test the hypothesis of independence, but this is outside the scope of the current paper and tool.*

Minor concerns:

8.	Authors should point out that their perturbation approach is to identify significant differences between region sets rather than significant overlap (i.e. the null hypothesis is that they are the same). Both hypotheses are of interest for different purposes, but the difference between them may not be clear to many readers.

*This is an excellent point, and one that provides a useful context on which to interpret our earlier Fisher’s exact test results. We have added some text to address this in the manuscript.*

9.	The authors state “once enough regions have been dropped, then adding has an increased prob-ability to add back a region that was previously dropped, causing a canceling effect.” While this is theoretically true, the probability of adding a random region of the genome that’s the same as one dropped is virtually nil. It would not make enough difference to be observable in the results. Rather than showing a defect in the Jaccard score, this seems to point to a problem in how Bedshift was written or implemented.

*Indeed, you rightly point out that the way we described adding regions is a virtually nil chance. Our statement was incorrectly written, and we have corrected this – in fact, the way Bedshift is implemented, the add perturbation comes before the drop perturbation, so the drop pertur-bation may then remove regions that were just added. So in fact it turns out not to be a defect in the Jaccard score or a problem with bedshift, but just an oversight on our part in terms of order of operations. In our re-run analysis, these issues have been clarified, and this point has become more understandable with the new universe analysis.*

10.	The parameters used for the shifting in the paper are not provided

*The default parameters were used for both adding and shifting. We have updated the methods section with this information.*

11.	Typo on page 1, line 60 (should be “calculate”)

*This typo has been fixed.*

Reviewer #2

Reviewer #2: Gu et al present a very interesting short report entitled “Bedshift: perturbation of genomic interval sets”. The content appears two-fold: one the one hand, they introduce bedshift, a novel python suite dedicated to randomly perturbing genomic coordinates; and on the other hand the authors carry out a systematic assessment work on four similarity metrics used to compare genomic intervals . The manuscript is well written and easy to read, the various sections are well described. The package per se is easy to install, well-documented on github as well as on the command line, and available from the shell inferface or python.

The work presented there is, in my opinion, far reaching. From what I could extrapolate, bedshift itself represents a much-needed tool that could be used systematically to quantify the significance of overlaps between genomic coordinates from control versus treatment conditions, by randomising the control. Out of experience, two conditions could easily represent such randomised versions of each other; I think this is where this tool comes in. It could also be used to determine random variability between replicates. The report further sheds light on candidate metrics to use depending on the type of experiment. For example, the shift method could simulate sliding, while the add method could represent external stimulation and the drop method the removal thereof or inhibition.

*Thank you for the comments, we appreciate the positive feedback! Indeed, we view this paper as presenting two independently useful advances: First, the bedshift software itself; and sec¬ond, an assessment of four similarity metrics, which provides an initial demonstration of the type of things that beshift can be used to do, and already leads to some interesting insights.*

I only have one concern and some minor comments but I think that the manuscript would benefit from addressing those:

1) Unless I missed it, in terms of reproducibility, did the authors observe similar results (i.e. metric scores) using other bed files chosen at random from ENCODE? It would be useful to have error bars on the metric graphs.

*This point was also raised by reviewer 1 (point #2). To address this, we re-ran the analysis using 3 different files of different types. We show how the metrics behave differently with different input files in figure S2. The general conclusions of the primary analysis are intact across files. In this case, we decided that error bars wouldn’t make sense, because the actual metrics are very different in terms of starting point on the different files, and it makes more sense to compare the plots for each file side-by-side, so we’ve displayed the data in this way in the supplemental figure.*

2)	As stated above, bedshift could be used as a very powerful randomisation control when compar-ing e.g. bed files from same extraction method (e.g. same antibody for ChIP-Seq) in two different conditions. I think the manuscript would substantially gain if the introduction and discussion clearly mentioned that it can be used to determine if e.g. variability between two conditions is random or biological. By this, I mean that it could be used by the scientific community to randomise a control file, and then computing e.g. the significance of overlap or any other metric between the randomised control and treatment

*Thank you for your comment on the practical applications of Bedshift. We have added more remarks on this topic in the Introduction section.*

3)	Page 2, lines 47-49, “These parameters were chosen arbitrarily to be not too small as to not have significant change, and not too large as to be completely different”.-Further to the previous point, I think it would be useful to present some level of significance associated with the metrics, e.g. with hypergeometric tests.

*This is a good point, and we agree that our text was a bit unsatisfying for describing how we chose the parameters. We’ve thought now a lot about how to best express this, and the significance levels proposed are problematic, since those tests are precisely the metrics we are comparing among the perturbed sets, and are a step removed from the known perturbation levels; so we thought a more intuitive way to convey this to visualize what the results of these perturbations look like. To address this, we’ve now added a new figure panel (1B), which depicts an example of a region set with 10 perturbations for each of the Low, Medium, and High perturbation levels. In the supplement, you can now find a new figure showing how these vary across all the perturbations. This figure does a good job of helping you intuit about what the bedshift operations do to an interval set. For convenience, we reproduce this figure below as Figure R1.*

4)	From the package documentation, bedshift -s and -a seem to present mean and stdev shift and add options. From the materials and methods, it is unclear by how much regions are shifted and added in analysis. Adding full settings of perturbations, with values for e.g. –shiftmean, –shiftstdev, – addmean, –addstdev in a supplementary methods section would benefit the study in terms of clarity. The detailed parameters of bedtools coverage should also be included. Also the details of overlap methods used to compute the Jaccard similarity index could be useful to have, i.e. were overlaps defined as total or partial overlap for a given region, if partial what was the minimum allowed?

*Indeed, we did not include sufficient detail in the methods, sorry about that. We have revised our paper to include a “Bedshift operations” section in the Methods, which details the pertur-bations and how they function with their parameters. We have also included the BedTools coverage parameters that we used in our similarity score. We reported percentage coverage for each region in the BED file it was called on. The overlap threshold for the Jaccard similarity index was mentioned in the Methods section for Jaccard score; regions having at least one base pair overlap were considered overlapping.*

5)	In the shift method, did the authors take into account the average distance between regions, as excessive shift could result in overlap if greater than the average distance between regions?

*Yes, this is an interesting point, and one that could definitely be tested with bedshift! The analysis would be to take files with different distances between regions and explore how the metrics behave. In our existing study, though, the files we chose are all quite sparse, so given the sparsity of our input bed files and the shift distance normal distribution that we used (0,150), it is unlikely that shifted regions overlap other regions. The shifted regions are more likely to overlap itself, which was by our design because we wanted to see if the similarity scores could detect a difference when regions overlapped as well as not overlapped.*

6)	Page 4, column 2, lines 25-26 “was constructed by merging regions from 300 BED files in the ENCODE dataset”. Can the authors comment on why they chose 300 experiments from ENCODE as universe and not e.g. the ENCODE regulatory database, or O’RegAnno or ReMap to name a few? Also, if they could clarify whether these were chosen randomly or not, and if not, what kind of experiments were chosen (TF ChIP-Seq, DNase-Seq, or ATAC-Seq?). Ideally they should not include broad histone peaks as these are very large.

*Indeed, this is something we have considered extensively and so we appreciate the opportu-nity to comment on this. The decision of universe is not an easy one. In the original paper, we wanted the universe to be tailored specifically to the current dataset, but the question of fit of universe definitely a major question, and is exactly the kind of question that we built bedshift for. To address this more comprehensively, in our revamped analysis, we included a study of varying universes. In the revision, we elected to use the universe from the SCREEN analysis of regulatory elements in ENCODE datasets, so that it’s a well-accepted universe. We then re-ran the entire analysis of 27 perturbation parameter sets using 3 additional universes, also from the SCREEN database. This analysis revealed that the fit of the universe is critical for the universe-based metrics, though less important for the non-universe-based metrics. This led to several new results and discussion points, and a new supplementary figure, which have been added to the paper.*

7)	On figure 2 and the associated methods, I could not determine how the combinations of operations were carried out, for example for shift+drop, is the drop carried out before or after shifting (I would assume after, but could not find confirmation in the text), could the authors clarify?

*We apologize for the confusion. The order of the operations was mentioned in the Overview of Bedshift section, but not in the methods. We have repeated this now in the “Bedshift opera¬tions” section in Methods to clarify. The order is shift, add, cut, merge, and drop. Furthermore, with our new file-based parameterization, the order can be selected by the user.*

8)	While most bed files will be unstranded, the rising number of e.g. CLIP/FLASH-Seq (which can be stranded) experiments as well as of e.g. eRNA or repeat element genomic regions (also potentially stranded) might warrant the implementation of a strandedness parameter in bedshift; alternatively the authors could discuss this limitation.

*This is an excellent point, and indeed, as it is implemented, Bedshift is not considering strand at all. We have discussed this limitation and added a “Future Development” section to the paper as well. As the files and generic similarity metrics we have been considering are all not stranded, this is not something we had considered.*

9)	Add+dropH and drop+addH seem to show recovering jaccard distance: this implies that regions added at random would partially reconstitute the original file. Is this plausible? And if so could this be prevented via a parameter?

*Excellent observation, and this point was also raised by reviewer one. Upon more detailed inspection, we realized that this is caused by the order of operations, which is add then drop – meaning that some of the added regions could be dropped. We have clarified this in the text. We also have a solution for this: the user can specify the order of perturbations using the Python API, or the new yaml-file configuration approach. This way, users can specify that drop should come before add, which will avoid this issue. This issue has also disappeared in our main analysis with the re-run, and instead, we comment on this possibility of increasing similarity with increasing pertubation, which shows up in our universe analysis for Euclidean distance. This is a very interesting situation that highlights the importance of choice of uni¬verse.*

10) For the shift method, the authors indeed mention that decreased coverage is expected, however can they further comment on why does add+shiftH result in increased coverage but add+drop and drop+add result in increased jaccard distance?

*Thank you for your question on this point. In our re-run analysis, this anomaly is no longer present in the primary analysis. Instead, a similar anomaly shows up in Euclidean distance in our universe analysis. We explain it in the paper like this:*

*In Scenario 6, we observe an interesting anomaly, that for the Euclidean distance, the slope turns positive, indicating that when adding regions is held constant, dropping more regions actually increases similarity between the files. This counterintuitive result can be explained by the possibility of dropping regions that were just added. In most cases, this is not a problem because the probability of dropping regions that were just added is low; however, in very specific situations, such as this particular scenario in our study, this effect can occur. The effect is most pronounced when universes are very different from the query file, because the add operation is adding regions unlikely to be in the original file, making it more likely that drop will drop something that’s different from the original file, increasing similarity. Also, it makes sense that this is more pronounced when add is higher, because it increases probability of dropping a region that is different. Further, we can explain that it only occurs for Euclidean distance, not for cosine distance, because for widely divergent universes, when adding lots of new unrelated dimensions, cosine distance is unaffected, as the angle is only calculated on the basis of dimensions present; in contrast, Euclidean distance explodes. This result emphasizes the importance of choosing an appropriate, fitting universe for vector-based approaches; if the universe is not a good reflection of the underlying data, then distance metrics may potentially behave counterintuitively, particularly for the Euclidean distance.*

**Second round of review**

**Reviewer 1**

Comparing the original version to this revision of Bedshift was like night and day - what an improvement! I really appreciate all the effort that the authors made to more clearly explain several points that were previously missing or written in a confusing way. I also appreciate the additional features added and the tests performed that helped flesh out what was happening. I agree to the removal of the FET results – this just would have confused readers, because the hypothesis is different.

The reviewers have nicely responded to all of my main concerns. I would not have used the original version of Bedshift, but can now think of ways it could be useful in my own lab’s research.

Only a few minor typos I noticed below:

Page 2, ~Line 43: conrast contrast

Page 3, line 4: order of perturbations

Page 3, line 26: universe is the unified set

**Reviewer 2**

The authors have answered several comments adequately and have appropriately added functionality where needed. The added figures have increased clarity. There is however a small number outstanding issues that I can see:

1) Re my previous point 1), while it is very useful to now detail the results using three classes of data (H3K4me3 ChIP-Seq, CTCF ChIP-Seq, DNase-Seq), it was to be expected that error bars/boxplots would not work comparing these three types or data; the comment aimed at producing a metric for reproducibility using similar data types. What the authors should do is to use e.g. at least 3 similarly-sized CTCF ChIP-Seq, H3K4me3 and DNaseI bed files (e.g. from related cell types) and attempt to produce error bars or overlay the metric scores for each "replicate". Alternatively this might also be derived using independent rounds of perturbations of the same file.

2) Based on the new manuscript, the term "DNase-H3K4me3" (page 3, lines 41-42) is I think confusing when referring to DHSs that show H3K4me3 signal if this is what the authors meant (for reference an H3K4me3 ChIP performed on MNase-digested chromatin can be called MNase-H3K4me3) so I suggest using the term DHS/H3K4me3 or DHS+/H3K4me3+

3) In the new section describing shift, page 6, lines 8-9 the authors write "a .fasta file containing chromosome lengths". I think this should read "a genome file containing chromosome lengths" as users will be familiar with this option (e.g. -g in bedtools slop); a fasta file also usually contains sequences.
